# Improvement of chemosensitivity and inhibition of migration via targeting tumor epithelial-to-mesenchymal transition cells by ADH-1-modified liposomes

**DOI:** 10.1080/10717544.2017.1417511

**Published:** 2017-12-20

**Authors:** Zhaoming Guo, Wenqing Li, Yue Yuan, Kun Zheng, Yu Tang, Kun Ma, Changhao Cui, Li Wang, Bing He, Qiang Zhang

**Affiliations:** ^a^ School of Life Science and Medicine, Dalian University of Technology Panjin Liaoning China; ^b^ State Key Laboratory of Natural and Biomimetic Drugs, School of Pharmaceutical Sciences, Peking University Beijing China

**Keywords:** Chemoresistance, migration, targeting, delivery, liposomes

## Abstract

How to overcome drug resistance and prevent tumor metastasis is key to the success of malignant tumor therapy. In this paper, ADH-1 peptide-modified liposomes (A-LP) have been successfully constructed for restoring chemosensitivity and suppressing cancer cell migration. With a particle size of about 90 nm, this functionalized nanocarrier was loaded with fluorescent probe or paclitaxel (PTX). Cellular uptake studies showed that A-LP facilitated the delivery of anticancer drug to tumor cells undergoing EMT. Interestingly, this nanocarrier enhanced chemosensitivity by assessing the cell activity using CCK-8 assay. Further, the results of Wound scratch assay and Transwell migration assay showed the inhibition effect of this nanocarrier on tumor cell migration. Moreover, this nanocarrier exhibited significant tumor-targeting ability and anti-tumor efficacy *in vivo*. Collectively, A-LP might be a novel targeted drug delivery system to enhance the efficacy of chemotherapy and prevent tumor metastasis.

## Introduction

Drug resistance and metastasis are two major obstacles to successful tumor chemotherapy and cause 90% of cancer-associated death (Hanahan & Weinberg, 2000). These two properties of malignancy have been studied extensively, but most studies have proceeded along separate pathways. However, some evidence shows that chemoresistance and metastasis might be linked during the progression of malignant tumor (Kerbel, 1990; Su et al., 1993). Furthermore, Yang et al. demonstrated that multidrug resistant (MDR) MCF7 cells displayed increased invasiveness (Yang et al., 2003). Recently, the role of the epithelial-to-mesenchymal transition (EMT) in cancer progression has been widely studied. Many studies reported that the development of drug resistance in cancer cells is accompanied by a transition from an epithelial to a mesenchymal phenotype (Li et al., 2009). Further, the cells undergoing EMT are more likely to survive therapeutics and become the source of metastasis (Hugo et al., 2007; Voulgari & Pintzas, 2009; Geers et al., 2013; Ye et al., 2015; Qi et al., 2016). And only cells undergoing EMT exhibited promoted invasion and MDR (Li et al., 2007a,b). Therefore, tumor cells undergoing EMT targeted therapy will be a promising strategy to enhance chemotherapeutic effect and prevent metastasis.

EMT is a process initially observed during embryonic development. When tumor cells undergo EMT, they lose epithelial characteristics and obtain mesenchymal properties including morphologic alteration which is often accompanied by the dissolution of epithelial tight junction, loss of cell adhesion, down-regulated expression of some epithelial markers such as E-cadherin as well as the acquisition of mesenchymal cadherins, for example, upregulated vimentin and N-cadherin (Hazan et al., 2004; Qi et al., 2016). Mesenchymal cadherins have been reported to be one of the key components contributing to cell motility and invasiveness (De Wever et al., 2004; Halbleib & Nelson, 2006; Wheelock et al., 2008). Therefore, the suppression of the mesenchymal molecules associated with EMT such as N-cadherin could be an effective strategy for abolishing the EMT-triggering effect.

N-cadherin is a transmembrane glycoprotein which is often overexpressed in many types of cancers such as breast cancer and colorectal carcinoma and is investigated as an inducer of EMT (Hazan et al., 2000; Yang et al., 2016). Therefore, N-cadherin may be an ideal target for tumor cells undergoing EMT targeted therapy. ADH-1 (N-AC-*CHAVC*-NH_2_), a cyclic pentapeptide, is not only an effective antagonist of N-cadherin mediated adhesion and migration but could selectively and competitively bind to it (Augustine et al., 2008; Burden-Gulley et al., 2009). Hence, ADH-1 was selected here as a targeting motif for EMT cells targeted delivery as well as an antagonist for blocking N-cadherin.

Nanocarriers, because of the advantages of enhanced distribution in tumor sites and reduced side effects to normal tissues, have been widely used as targeted drug delivery vehicles (Wang et al., 2011; Xiang et al., 2011; Zhang et al., 2012; Li et al., 2016). Liposomes are the most promising application in clinical use because of their advantages in biocompatibility, improving drug solubility and stability as well as achieving favorable pharmacokinetic properties (Allen, 1998), such as doxorubicin liposomes (Doxil^®^). At present, nanocarriers modified with targeting ligands are developed to bind specifically to tumor cells and increase the endocytosis of drugs into cancer cells (Marcucci & Lefoulon, 2004). Although many attempts have been made, very limited success has been achieved in active targeting cancer therapy (Blanco et al., 2011). The EMT cells may account for the failure of nanomedicine and become a great challenge. However, few investigations on EMT cells targeted therapy have been performed to date.

In this paper, we constructed ADH-1-modified liposomes (A-LP) for tumor cells undergoing EMT targeted therapy. PTX-resistant MCF7 cells (MCF7 PTX-R) were used here as a model of EMT cells. We hypothesized that A-LP could effectively reverse chemoresistance and inhibit tumor cell migration through the following steps: first, A-LP are specifically recognized by tumor cells undergoing EMT via the specific interaction between ADH-1 and N-cadherin. Second, ADH-1 suppressed the EMT process by blocking the function of N-cadherin. Finally, the encapsulated PTX further kills tumor cells.

## Materials and methods

### Materials

DSPE-PEG_2000_-NHS and DSPE-PEG_2000_ were purchased from NOF Corporation (Tokyo, Japan). Egg phosphatidylcholine (EPC) was obtained from Pharmacia Biotech (Piscataway, NJ, USA) and cholesterol (Chol) was obtained from Lipoid GmbH (Ludwigshafen, Germany). Paclitaxel (PTX) was purchased from Haikou Pharmaceutical Co., Ltd. (Hainan, China). DAPI, Cell counting kit-8 (CCK-8) and secondary antibody labeled with FITC were purchased from Beyotime Biotechnology Co., Ltd. (Shanghai, China) and Coumarin-6 was purchased from Shanghai Aladdin Bio-chem Technology Co., Ltd. (Shanghai, China). The modified ADH-1 peptide (CHAVC)-NH_2_ (Mw 528.66) was synthesized (purity 95%) by GL Biochem Peptide Co., Ltd. (Shanghai, China). Sephadex G-50 was from Pharmacia Biotech (Piscataway, NJ). RIPA Lysis Buffer, PMSF and enhanced chemiluminescence kit were purchased from Coolaber Science and Technology Co., Ltd. BCA Kit was obtained from Solarbio Science and Technology Co., Ltd. (Beijing, China). All antibodies were obtained from Biodragon-immunotech (Beijing, China). Transwell filter and matrigel were purchased from BD Biosciences (USA). All other chemicals were of analytical grade and used without further purification. Taxol was commercially available from the hospital of Beijing (Bristol-Myers Squibb Co., Princeton, NJ), containing 30 mg of paclitaxel in a 5.0 mL mixture of Cremophor EL and ethanol (1:1, v/v).

MCF7 human breast cancer cell line were purchased from the Institute of Basic Medical Science, Chinese Academy of Medical Science (Beijing, China). Cells were cultured in RPMI-1640 (HyClone) with 10% fetal bovine serum (FBS) and antibiotics (100 U/mL penicillin and 100 mg/mL streptomycin) at 37 °C in 5% CO_2_.

Female BALB/c nude mice (18–22 g) were supplied by Peking University Health Science Center (Beijing, China). During the experiments, mice were kept under SPF condition with free access to standard food and water. All the *in vivo* experiments were performed with the guidelines approved by the Institutional Animal Care and Use Committee of Peking University.

### Preparation of ADH-1-modified liposomes

In order to prepare ADH-1-modified liposomes (A-LP), the ADH-1 peptide was conjugated to DSPE-PEG_2000_-NHS according to a previous method (Guo et al., 2014). Briefly, DSPE-PEG_2000_-NHS dissolved in DMSO was added to ADH-1 dissolved in DMSO at 2:1 molar ratio, adjusting pH to 8.0 with triethylamine. The reaction proceeded for 3 days under moderate stirring at room temperature and monitored by reversed phase high-performance liquid chromatography (Thermo Fisher, UltiMate3000, MA, USA) at 220 nm. The mobile phase consists of (A) water containing 0.1% trifluoroacetic acid and (B) acetonitrile containing 0.1% trifluoroacetic acid. The gradient elution was 8%–33% (B) in 15 min and the flow rate was 1 mL/min. The ADH-1 peptide was detected at 220 nm. Then, the reaction mixture was dialyzed (molecular mass cut off 3500) against deionized water for 48 h to remove the unconjugated peptide and the solvent DMSO. The final solution was lyophilized and stored at −20 °C. The conjugation of ADH-1-PEG-DSPE was confirmed using a matrix-assisted laser desorption/ionization-time of flight (MALDI-TOF) mass spectrometer (Bruker Daltonics, Germany).

Liposomes were prepared by a film dispersion method (Guo et al., 2014). A-LP was composed of EPC, cholesterol, DSPE-PEG and ADH-1-PEG-DSPE (65:20:4.35:0.435, mol/mol). LP was composed of EPC, cholesterol and DSPE-PEG (65:20:4.785, mol/mol). To prepare fluorescent-labeled liposomes for cellular uptake investigation, a hydrophobic fluorescent probe, coumarin-6 (lipids: coumarin-6 = 22600: 9, w/w) was loaded into liposomes. Briefly, the lipids and coumarin-6 were co-dissolved in chloroform/methanol (2:1, v/v) mixture and evaporated at 37 °C under reduced pressure. Then the thin film was hydrated with phosphate-buffered saline (PBS, pH 7.4). Afterwards, the suspensions were treated by an ultrasonic cell disruptor for 2 min (Amplitude: 10). The unencapsulated coumarin-6 was separated by a Sephadex G-50 column. For cytotoxicity studies, PTX (lipids: PTX = 22.6:1) loaded liposomes were prepared with the same method as above.

### Characterization of liposomes

The particle size and surface charge of liposomes were measured by dynamic light scattering (DLS) analysis using Malvern Zetasizer Nano ZS (Malvern, UK) at 25 °C. The morphology of liposomes was observed by transmission electron microscope (TEM) after negative staining with 1% phosphotungstic acid solution.

Concentration of coumarin-6 was determined by fluorophotometer (Hitachi, F-7000, Japan). The excitation wavelength and emission wavelength were set at 467 nm and 502 nm, respectively. PTX was quantified by a HPLC system using a C18 column and a mobile phase containing methanol, acetonitrile and water (40:30:30, v/v/v) at a flow rate of 1.0 mL/min, and detected at 227 nm. The encapsulation efficiency (EE) of coumarin-6/PTX was calculated as coumarin-6/PTX loaded in the liposomes divided by total coumarin-6/PTX used.

The *in vitro* coumarin-6 leakage from liposomes was measured by a dialysis method. Briefly, the coumarin-6-loaded liposome solutions were placed in the dialysis bags (*M*_w_ cutoff of 14,000 Da) and dialyzed against PBS at 37 °C under horizontal shaking (100 rpm). At predetermined time points, aliquots were withdrawn and replaced with equal volume of PBS. The coumarin-6 content was measured by fluorophotometer as described above.

### Establishment of PTX-resistant MCF7 cells

To establish stable PTX-resistant MCF7 cells (MCF7 PTX-R), MCF7 cells were continuously exposed to PTX for more than 5 months. In the course of cell culture, the medium was replaced every 3 days with a stepwise increase of paclitaxel concentration, 2-fold increase at each step of resistance, from 2 nM up to 32 nM. Gradually, these cells displayed resistance to PTX and then, the cells were further cultured for a month in the medium containing PTX before characterization studies.

### Morphologic observation

Cells were grown to 70% confluence in RPMI-1640 plus 10% FBS (MCF7 parental cells) or RPMI-1640 plus 10% FBS plus 32 nM PTX (MCF7 PTX-R cells) and observed at ×10 magnification with inverted microscope (Leica DMI4000B). The images of the MCF7 PTX-R cells and parental cells were compared for morphologic characteristics consistent with EMT [i.e. spindle shaped cells and increase in intercellular separation].

### Western blot assay

Western blotting was performed to study the change of the protein expression. Cells treated with different formulations were lysed in RIPA Lysis Buffer and PMSF cocktail (final concentration of PMSF is 1 mM). Then, lysates were centrifuged at 13,000 g/min for 5 min and supernatants were collected. The proteins were quantitated using the BCA Kit and separated by SDS-polyacylamide gels and then transferred to the PVDF membranes. The PVDF membranes were incubated with the indicated primary antibody at 4 °C overnight. Subsequently, the membranes were incubated with HRP Conjugated Polyclonal Goat Anti-Rabbit IgG (H + L) (1:8000) for 1.5 h at the room temperature. The immune complexes were visualized using an enhanced chemiluminescence kit on sensitive chemiluminescent imaging (FluoChem HD2, Protein Simple, USA).

### Fluorescent immunohistochemistry

MCF7 Parental and PTX-R cells were grown on coverslips for 24 h at 37 °C. The cells were rinsed with cold PBS for twice, fixed with 4% paraformaldehyde, blocked with 5% BSA for 1 h at room temperature and incubated with mouse monoclonal antibody to N-cadherin (BD-M10211, Promab). Negative controls (PBS added) were included. A FITC-conjugated Goat Anti-mouse IgG was used as secondary antibody and incubated with cells for 1 h at room temperature. Cell nuclei were stained with DAPI, and the samples were observed using a Leica TCS SP8 confocal laser-scanning microscope (CLSM, Heidelberg, Germany).

### Cellular uptake studies

#### Confocal microscopy analysis

MCF7 Parental and PTX-R cells were grown on coverslips for 24 h. Then, the cells were incubated with coumarin-6 loaded A-LP or LP (containing 100 ng/mL coumarin-6) in serum-free medium. For the receptor competition experiments, cells were incubated with free ADH-1 (2 μM) for 1 h at 37 °C prior to the addition of coumarin-6-loaded A-LP. After 3-h incubation of each liposome formulation at 37 °C, the medium was removed and cells were washed with cold PBS three times, fixed with 4% paraformaldehyde (v/v) at room temperature for 20 min, followed by cell nuclei staining with DAPI for 15 min. Then, cells were imaged using a laser scanning confocal microscope (LSCM, Leica, TCS SP8, Germany).

#### Flow cytometry analysis

MCF7 Parental and PTX-R cells were seeded into 12-well plates and cultured for 24 h at 37 °C, respectively. Then cells were treated with coumarin-6-loaded A-LP or LP (containing 100 ng/mL coumarin-6) in serum-free medium. For the receptor competition experiments, cells were incubated with free ADH-1 (2 μM) for 1 h at 37 °C prior to the addition of coumarin-6-loaded A-LP. After 3-h incubation of each liposome formulation at 37 °C, the cells were rinsed with cold PBS (pH = 7.4) twice, then detached by trypsinization and suspended in 400 μL of PBS. The samples were analyzed by flow cytometry using the FACScan flow cytometer (Becton Dickinson FACSCalibur, USA).

### Drug sensitivity assay

MCF7 PTX-R cells were cultured in a 96-well plate at a density of 5000 cells per well for 24 h. Then, the cells were incubated with fresh medium containing serial concentrations of LP/PTX, A-LP/PTX and free PTX (1-96 nM). After further incubation for 48 h at 37 °C, 10 μL of CCK-8 solution was added into each well. The cells were incubated for 1 h at 37 °C, and then, the absorbance was determined using a 96-well plate reader (BioTek, Synergy H1). All data were shown as the percentages of viable cells relative to the survival of control group (cells treated with medium).

### Wound scratch assay

MCF7 PTX-R cells were seeded into a 6-well plate. When the cells became confluent, cells were disrupted with a uniform scratch using a 200-μL pipette tip, rinsed with cold PBS three times to remove free-floating cells and debris. Then, the cells were incubated in culture medium containing LP/PTX, A-LP/PTX, A/PTX (preincubated with free ADH-1 for 1 h before treated with PTX) (containing 96 nM PTX) or free ADH-1 alone for 24 h and observed at ×5 magnification with inverted microscope (Leica DMI4000B). The groups of A/PTX and A-LP/PTX were added to make the final concentration of ADH-1 at 2 μM. Quantitative analysis of relative mobility shown as percentage was calculated using 10 randomly chosen distances across the wound at 0 h and 24 h, divided by the distance measured at 0 h.

### Migration assay

The Transwell migration assay was used to evaluate the migration inhibition of LP/PTX, A-LP/PTX, free ADH-1 before treated with PTX (A/PTX) or free ADH-1 alone according to a previous method (Qin et al., 2014). Briefly, the upper chambers with polycarbonate membranes containing 8 μm pores were washed with PBS and incubated in 1% bovine serum albumin (BSA) for 1 h. MCF-7 PTX-R cells were seeded into the upper chamber of the transwell, 5% fetal bovine serum was placed in the lower chamber as attractant. Then, the cells were allowed to grow at 37 °C in serum-free medium. An equal volume of LP/PTX or PBS was added as a compared group and a blank control group. After 24 h, the upper chambers were washed with PBS and the cells on the upper surface were scrubbed off with humid cotton buds. Then, the cells on the bottom surface were fixed with methanol for 20 min and stained with 0.1% crystal violet solution for 30 min. Number of cells that migrated through the polycarbonate membrane was counted in six fields with an optical microscope. The inhibition rate was calculated compared with blank control group.

### *In vivo* distribution of DiR-loaded a-LP by living fluorescence imaging

The tumor model was established by subcutaneous inoculation of 4 × 10^6^ MCF7 PTX-R cells in the right flanks of female BALB/c nude mice. When the tumor volume reached approximately 500 mm^3^, mice were randomly divided into two groups (3 per group), treated with 0.2 mL of the formulations by tail vein injection. The concentration of DiR was 1.5 mg/mL. At predetermined time points, each group was anesthetized with isoflurane and photographed with Kodak In Vivo Imaging System FX PRO (Carestream Health, Inc.,). After 48 h, the mice were sacrificed.

### *In vivo* antitumor efficacy

The tumor model was established as described above. When the tumor volume reached approximately 40–50 mm^3^ on the 5th day, the mice were randomly divided into four groups (*n* = 4). Each group received 0.2 mL of saline, Taxol, LP(PTX) or A-LP (PTX) via the tail vein at a dosage of 10 mg PTX/kg body weight every other day for a total of four times. Tumor volumes were measured with a caliper every other day. Tumor volume was calculated as: [length × (width)^2^]/2. At the end of the experiment, the mice were sacrificed and the tumors were excised.

### Statistical analysis

All the quantitative data are shown as means ± SD. Student’s *t-*test was performed in statistical analysis. The difference between two groups was considered statistically significant with a *p* value less .05, and highly significant with a *p* value less than .01.

## Results and discussion

### Preparation and characterization of a-LP

**Figure 1.**
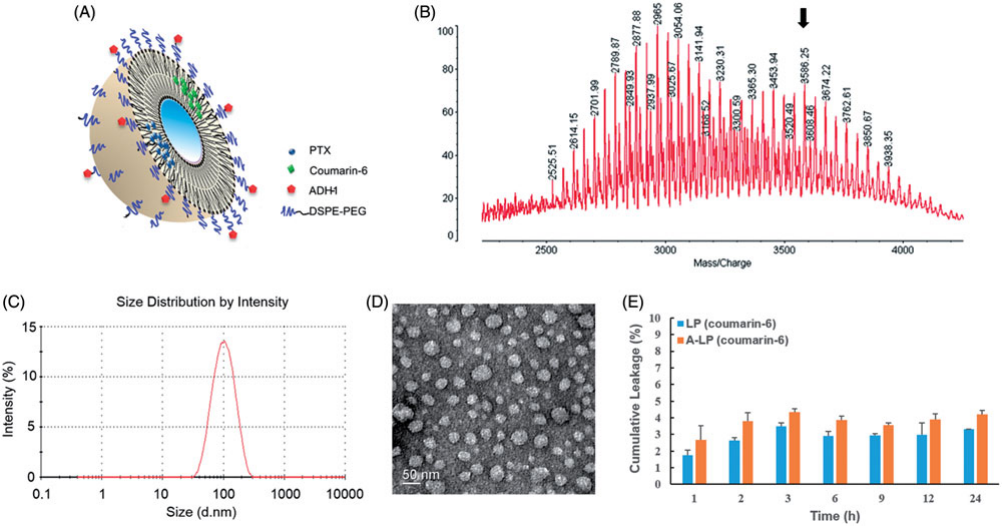
The characteristics of ADH-1-modified liposomes (A-LP). (A) Schematic illustration of A-LP. (B) MALDI-TOF MS spectra of ADH-1-PEG-DSPE. The arrow indicates the peak of ADH-1-PEG-DSPE. (C) Size distribution graph of A-LP by dynamic light scattering analysis. (D) Transmission electron microscopy image of A-LP. Scale bar is 50 nm. (E) *In vitro* cumulative leakage profile of coumarin-6 from liposomes in PBS at 37 °C at 100 rpm (mean ± SD, *n* = 3).

A schematic representation of A-LP loaded with fluorescent probe or PTX was shown in Figure 1(A). First, the targeting material DSPE-PEG-ADH-1 was prepared by a nucleophilic substitution reaction between the NHS group of DPSE-PEG-NHS and the terminal amino group of the ADH-1 peptide. MALDI-TOF MS data validated the successful synthesis of DPSE-PEG-ADH-1, as the experimental molecular weight (MW) of DPSE-PEG-ADH-1 was observed to be approximately 3500 Da, which was in accordance with the theoretical calculated MW (Figure 1(B)). The unreacted DSPE-PEG-NHS turned into DSPE-PEG (the peak of MW 3054 Da) during the dialysis process due to the NHS group was hydrolyzed and used as normal DSPE-PEG in the preparation of liposomes. The conjugation efficiency was more than 90% monitored by HPLC (data not shown).

Then, the various liposomes loaded with coumarin-6 or PTX were prepared and their particle size, zeta potential and encapsulation efficiency (EE) were summarized in Table S1. The particle sizes of different liposomes were similar, approximately 90 nm, with a narrow size distribution (PDI <0.18). All the liposomal formulations exhibited slightly surface charges. No significant difference in particle size and surface charge was detected in the various liposomes mentioned in this study. A typical particle size and distribution graph of A-LP were shown in Figure 1(C) and the spherical morphology of A-LP was shown in the TEM image (Figure 1(D)). In addition, data showed that the EE of coumarin-6 or PTX was more than 95%.

The leakage of coumarin-6 from liposomes *in vitro* was assessed in PBS. As shown in Figure 1(E), less than 5% of coumarin-6 leaked out from A-LP or LP within 24 h. The incubation time of liposomes was 3 h in following cellular uptake experiments. Therefore, it could be predicted that most of coumarin-6 was uptake into cells in the form of liposomes and the fluorescence signal of coumarin-6 could indicate the behavior of liposomes.

### Acquisition of PTX resistance induces morphologic changes and specific protein changes consistent with EMT

**Figure 2.**
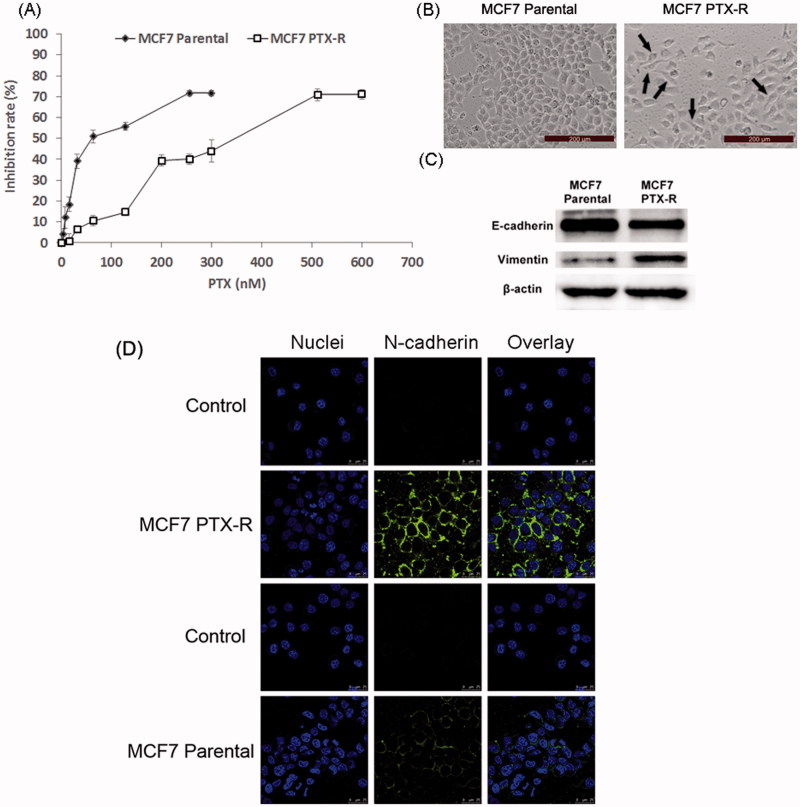
Acquisition of PTX resistance induces morphologic changes and specific protein changes consistent with EMT in MCF7 cells. (A) PTX-sensitivity assay in parental MCF7 and MCF7 PTX-R cells. IC50 values of parental MCF7 and MCF7 PTX-R cells were 77.7 nM and 320.7 nM, respectively. (B) Cell morphology of parental MCF7 and MCF7 PTX-R cells was observed by microscopy at ×10 magnification. The parental MCF7 cells had an epithelioid and paving stone appearance. In contrast, MCF-7 PTX-R cells (black arrows) adopted spindle-shaped or pear-like morphology and showed a decrease in cell-cell contacts. (C) The expression of EMT-related markers in parental MCF7 and MCF7 PTX-R cells. (D) MCF7 PTX-R cells exhibit increased expression of N-cadherin located in cell membrane. Immunofluorescence staining for N-cadherin was done on MCF7 PTX-R cells and parental cells. Cell nuclei were stained with DAPI.

MCF7 PTX-R cells were successfully established after continuous exposure to PTX for more than five months. Figure 2(A) showed the PTX-sensitivity assay of parental MCF7 and MCF7 PTX-R cells. The IC50 values of parental MCF7 and MCF-7 PTX-R cells were 77.7 nM and 320.7 nM, respectively. We could calculate the resistance index was 4.13. These results demonstrated that MCF7 PTX-R cells obtained PTX resistance. In addition, MCF7 PTX-R cells showed increased resistance to doxorubicin (Figure S1). MCF7 PTX-R cells displayed markedly different light-microscopic appearance compared with the parental cells (Figure 2(B)). The parental MCF7 cells had an epithelioid and paving stone appearance. In contrast, MCF-7 PTX-R cells adopted spindle-shaped or pear-like morphology and showed a decrease in cell–cell contacts. These changes were typical morphologic characteristics of the mesenchymal phenotype. Hence, MCF-7 PTX-R cells have transformed into the mesenchymal phenotype consistent with the morphologic characteristics of cells undergoing EMT (Yang et al., 2006; Nieto, 2009).

Besides, cells undergoing EMT lose their epithelial characteristics and instead take on mesenchymal properties including down-regulation of the epithelial cell marker E-cadherin and up-regulation of the mesenchymal cell markers express the mesenchymal markers such as vimentin and N-cadherin (Acloque et al., 2009; Thiery et al., 2009). To further verify the successful establishment of EMT cell model, Western blot assay was performed to determine the specific protein changes. Compared with parental MCF7 cells, MCF7 PTX-R cells showed increased expression of vimentin and reduced expression of E-cadherin (Figure 2(C)).

Then, we further measured the expression of N-cadherin by immunofluorescence method. As shown in Figure 2(D), compared with parental MCF7 cells, MCF7 PTX-R cells exhibited increased expression of N-cadherin located in cell membrane. This also suggested that N-cadherin could be a target for EMT cells selected delivery.

According to these results above, it could be concluded that the occurrence of EMT in MCF7 PTX-R cells.

### Targeted delivery to MCF7 PTX-R cells

**Figure 3.**
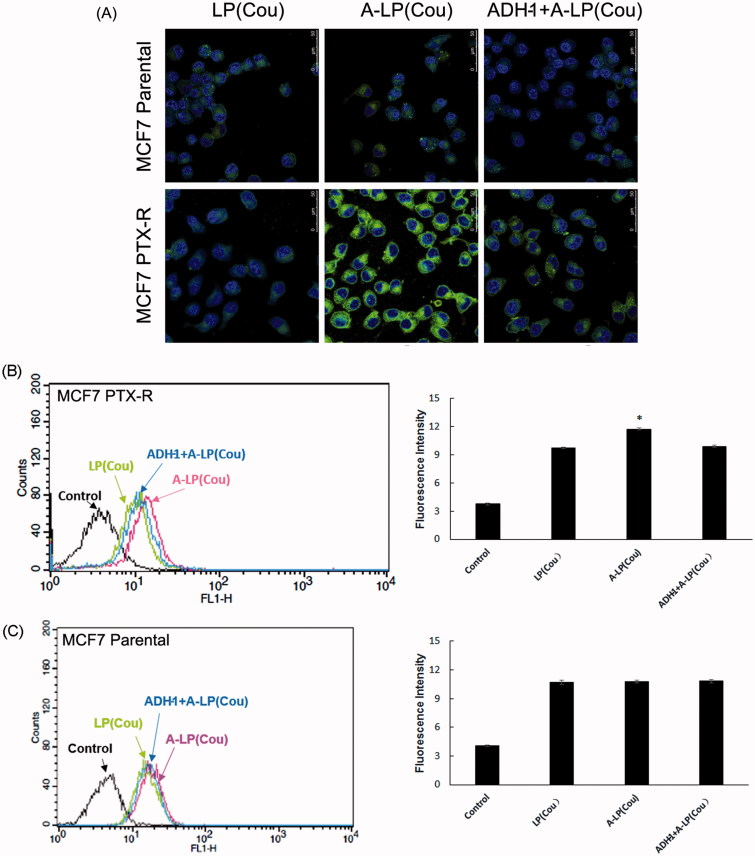
Targeted delivery to MCF7 PTX-R and parental cells by confocal microscopy analysis (A) and flow cytometry studies (B and C). Cells were treated with LP (cou), A-LP (cou) or A-LP (cou) preincubated with free ADH-1 (2 μM) for 1 h at 37 °C for 3 h. Data are presented as mean ± SD (*n* = 3). *p < .05, versus LP (cou) or A-LP (cou) pre-incubated with free ADH-1.

Figure 3(A) displayed the cellular fluorescence intensity of liposomes loaded with coumarin-6 in parental MCF7 and MCF7 PTX-R cells (laser confocal microscopy images). Obviously, the intracellular fluorescence intensity of coumarin-6 in A-LP group was remarkably higher than that in LP group. While in parental MCF7 cells, the cellular uptake had no significant difference between the A-LP-treated groups and LP-treated ones. We further investigated the mechanism of enhanced cellular uptake. After blocking the N-cadherin by preincubation with free ADH-1 peptide, the intracellular fluorescence intensity of coumarin-6 in A-LP group was markedly decreased. These results indicated that the enhanced cellular uptake of A-LP was mediated by N-cadherin in MCF7 PTX-R cells. Similar results were obtained by flow cytometry analysis (Figure 3(B,C)).

### Enhanced drug sensitivity in MCF7 PTX-R cells

**Figure 4.**
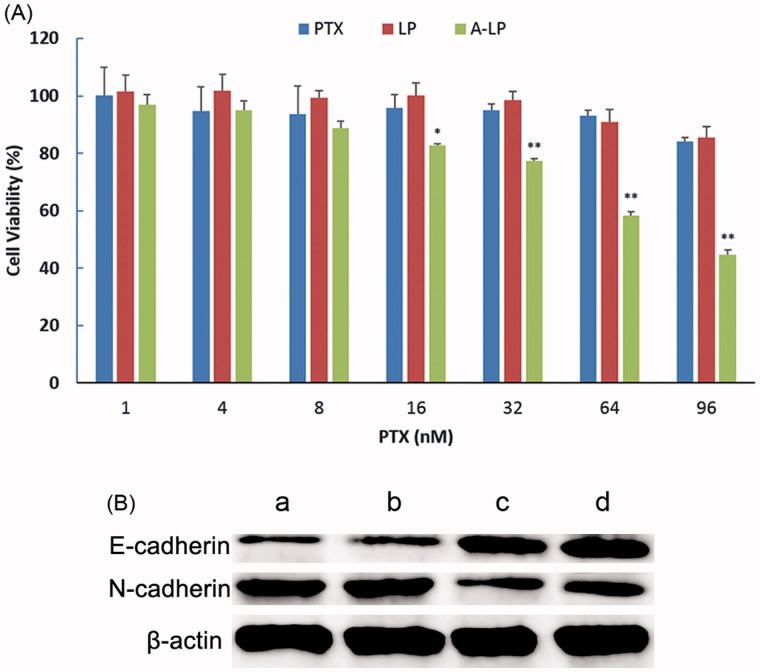
(A) Chemosensitivity evaluation of PTX-loaded A-LP in MCF7 PTX-R cells. MCF7 PTX-R cells were treated with free PTX, LP/PTX or A-LP/PTX for 48 h at 37 °C by CCK-8 assay. Each bar represents mean ± SD (*n* = 3). *p < .05, *vs* LP/PTX or free PTX; **p < .01, *vs* LP/PTX or free PTX. (B) A-LP/PTX suppresses the EMT process on MCF7 PTX-R cells. Changes in the protein expressions of the epithelial marker E-cadherin and the mesenchymal marker N-cadherin were analyzed by western blotting. β-actin was used as a loading control. MCF7 PTX-R cells were treated with (a) complete RPMI-1640, (b) LP/PTX, (c) A-LP/PTX, (d) ADH-1 for 24 h.

The sensitivity of MCF7 PTX-R cells to free PTX, PTX-loaded LP (LP/PTX) and A-LP (A-LP/PTX) was assessed using CCK-8 assay. As shown in Figure 4(A), after incubation of 48 h, the viability of MCF7 PTX-R cells treated with A-LP/PTX decreased markedly.However, for free PTX or LP/PTX group, the proliferation of cancer cells had little change. This further indicated that MCF7 PTX-R cells indeed exhibited PTX resistance. For A-LP/PTX, the enhanced proliferation inhibition to MCF7 PTX-R cells was probably attributed to improved cellular uptake and enhanced chemosensitivity due to the modification of ADH-1 peptide (Augustine et al., 2008). Because ADH-1 peptide could not only bind to N-cadherin but block it, the EMT process was suppressed to some extent. Changes in the protein expressions of the epithelial marker E-cadherin and the mesenchymal marker N-cadherin were analyzed by western blotting (Figure 4(B)). Compared with LP/PTX, A-LP/PTX suppresses the EMT process on MCF7 PTX-R cells as well as ADH-1. It is reported that EMT results in suppression of drug transporter and concentrating proteins, contributing to drug resistance (Zheng et al., 2015). Besides, the expression of Twist, which is a contributor to acquired paclitaxel resistance, is elevated in the EMT process (Wang et al., 2004; Cheng et al., 2007; Qi et al., 2016). Accordingly, suppression of EMT could reverse chemoresistance and improve sensitivity to chemotherapy. The cytotoxicity of ADH-1 to MCF7 PTX-R cells was evaluated (Figure S2) and the result showed that ADH-1 had no influence on the proliferation of MCF7 PTX-R cells.

### The effect of a-LP/PTX on migration inhibition

**Figure 5.**
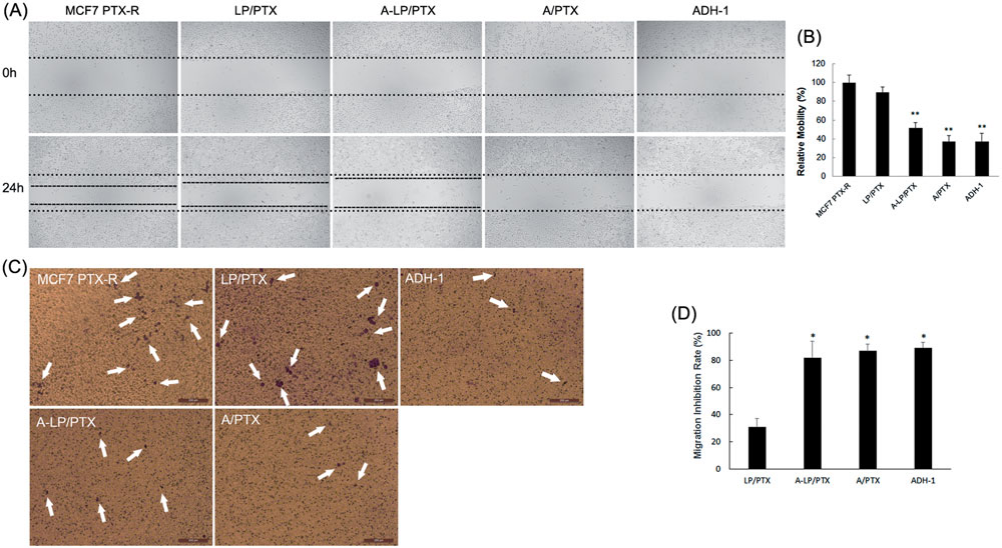
Migration inhibition effect of ADH-1-modified liposomes (A-LP/PTX) on MCF7 PTX-R cells. (A) Wound scratch assays were performed with a uniform scratch using a 200-μL pipette tip. Then, the cells were washed with culture media to remove any free-floating cells and debris and incubated in culture medium containing LP/PTX, A-LP/PTX, free ADH-1 (2 μM) before treated with PTX (A/PTX) or free ADH-1 (2 μM) alone for 24 h and observed at ×5 magnification with inverted microscope. (B) Relative motility was calculated using 10 randomly chosen distances across the wound at 0 h and 24 h. **p < .01, versus LP/PTX. (C) Optical images of cells (white arrows) on the bottom surface of the Transwell inserts after treatment with LP/PTX, A-LP/PTX, free ADH-1 (2 μM) before treated with PTX (A/PTX) or free ADH-1 (2 μM) alone for 24 h. (D) The inhibition rate was calculated by counting cells that migrated through polycarbonate membranes of the inserts. *p < .05, versus LP/PTX.

First, the migratory ability of parental MCF7 and MCF7 PTX-R cells was evaluated by Wound scratch assay and Transwell migration assay. As shown in Figure S3 and Figure 5, compared with parental MCF7 cells, MCF7 PTX-R cells showed a significantly enhanced capacity of migration. This result indicated that drug resistance and metastasis are linked during the progression of cancer.

Then, we performed wound scratch assay by scratching the cell layer prior to incubation with LP/PTX, A-LP/PTX, free ADH-1 before treated with PTX (A/PTX) and free ADH-1 alone. As shown in Figure 5(A,B), the mobility of MCF7 PTX-R cells was significantly inhibited by A/PTX and A-LP/PTX as well as ADH-1, whereas the nonmodified LP/PTX did not show any inhibition effect. Further, the result of the transwell migration assay also confirmed the effect of A-LP/PTX on migration inhibition. As shown in Figure 5(C), there was an obvious decrease in the number of cells migrating through polycarbonate membranes in ADH-1, A/PTX or A-LP/PTX group. The migration inhibition rate of A-LP/PTX group, A/PTX group or ADH-1 group was 2.5-fold of that of LP/PTX group (Figure 5(D)). Since ADH-1 could selectively and competitively binds to and blocks N-cadherin (Augustine et al., 2008) and it is reported that N-cadherin promotes cell migration during tissue morphogenesis (Halbleib & Nelson, 2006). Thus, the results above suggested that A-LP/PTX inhibited the migration of EMT cells owing to the modification of ADH-1 and the inhibition effect was probably mediated by N-cadherin. Migration is a necessary step in tumor metastasis (Chambers et al., 2002). Therefore, EMT targeting may be a promising strategy to prevent metastasis and the increased expression of N-cadherin in tumor cells undergoing EMT makes it possible.

### Targeting delivery and anti-tumor efficacy *in vivo*

**Figure 6.**
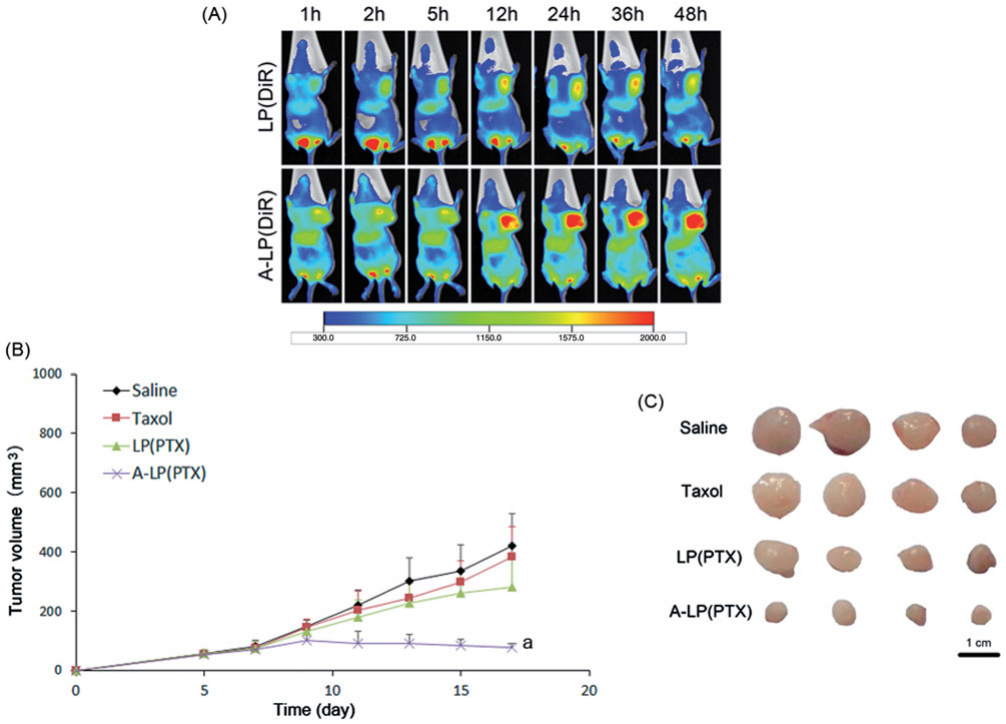
(A) *In vivo* fluorescent images of tumor bearing mice at different time points after i.v. injection of LP (DiR) or A-LP (DiR). (B) Tumor growth curve of MCF7 PTX-R cells baring nude mice treated with saline, Taxol, LP (PTX) or A-LP (PTX). a, *p* < .05 versus saline, Taxol or LP (PTX) formulation. (C) Photograph of the tumors excised at the end of the test. Scale bar =1 cm.

To further validate the targeting delivery of A-LP *in vivo*, we conducted living fluorescence imaging studies. Figure 6(A) showed the real-time distribution and tumor accumulation of DiR-loaded LP and A-LP in the MCF7 PTX-R-bearing mice at different time after iv injection. Both groups displayed specific distribution in tumor over time through passive targeting. However, the accumulation of DiR in A-LP (DiR) were much stronger and lasted longer at high level than that in LP (DiR) group. Besides, the fluorescent signal in LP (DiR) group decreased since 36 h, while the fluorescent signal in A-LP (DiR) group maintained at a high level until 48 h. These results were possibly due to the active targeting effect and the slow clearance of the active targeting liposomes (Guo et al., 2015).

Then, the *in vivo* anti-tumor efficacy of A-LP (PTX) was investigated in BALB/C nude mice bearing MCF7 PTX-R breast cancer xenografts. As shown in Figure 6(B), neither Taxol nor LP (PTX) was effective in inhibiting MCF7 PTX-R tumor growth compared with physiological saline. However, A-LP (PTX) showed significant inhibition on tumor growth during the test. Significant difference was observed between A-LP (PTX) and LP (PTX) (*p* < .05) or Taxol (*p* < .05). This result was consistent with its increased tumor accumulation and enhanced chemosensitivity. Similar result was found in tumor size at the end of test (Figure 6(C)).

## Conclusions

In this study, ADH-1 peptide modified liposomes for improving chemo-sensitivity and inhibiting tumor cells migration had been successfully established and characterized. A-LP had a uniform size distribution. In vitro studies, A-LP enhanced cellular uptake via specific recognition between ADH-1 and N-cadherin, and showed higher cytotoxicity to MCF7 PTX-R cells. Importantly, A-LP showed remarkable inhibition effect on tumor cell migration. Besides, A-LP increased accumulation in tumor tissue *in vivo*. Furthermore, this nanocarrier improved the efficacy of anticancer drug loaded for tumor undergoing EMT. In conclusion, this study might provide a novel strategy for antitumor therapy by targeting EMT cells and highlights the importance of combining EMT inhibition with chemotherapy.

## Supplementary Material

IDRD_Guo_et_al_Supplemetal_Content.docx
