# Supplementary material for: Improvement of chemosensitivity and inhibition of migration via targeting tumor epithelial-to-mesenchymal transition cells by ADH-1-modified liposomes
Source: Drug Deliv. 2017 Dec 20;25(1):112–21. doi: 10.1080/10717544.2017.1417511 (PMC6058515; doi:10.1080/10717544.2017.1417511)
Supplement: IDRD_Guo_et_al_Supplemetal_Content.docx [file IDRD_A_1417511_SM8677.docx]

Supporting Information

Improvement of Chemosensitivity and Inhibition of Migration via Targeting Tumour Epithelial-to-Mesenchymal Transition Cells by ADH-1-Modified Liposomes

Zhaoming Guo ^a,^ *, Wenqing Li ^a^, Yue Yuan ^a^, Kun Zheng ^a^, Yu Tang ^a^, Kun Ma ^a^, Changhao Cui ^a^, Li Wang ^a^, Bing He ^b^, Qiang Zhang ^b^

*^a^ School of Life Science and Medicine,* *Dalian University of Technology, Panjin, Liaoning 124221, China*

*^b^ State Key Laboratory of Natural and Biomimetic Drugs, School of Pharmaceutical Sciences, Peking University, Beijing 100191, China*

***Corresponding author：** School of Life Science and Medicine, Dalian University of Technology, Panjin, Liaoning 124221, China.

Tel: +86-427-2631427. Fax: +86-427-2631889. E-mail address: guozm@dlut.edu.cn (Zhaoming Guo).

Table S1. Characterization of the liposomes (n = 3)

| Formulations | Size (nm) | PDI | Zeta potential (mV) | EE (%) |
| --- | --- | --- | --- | --- |
| LP (Cou) | 88.58$\pm$1.21 | 0.174$\pm$0.011 | -2.92$\pm$0.42 | 99.03$\pm$1.32 |
| A-LP (Cou) | 90.09$\pm$1.42 | 0.155$\pm$0.013 | -3.12$\pm$0.31 | 99.24$\pm$1.56 |
| LP (PTX) | 89.43$\pm$0.98 | 0.161$\pm$0.021 | -3.10$\pm$0.33 | 99.30$\pm$0.96 |
| A-LP (PTX) | 89.93$\pm$1.46 | 0.154$\pm$0.017 | -3.92$\pm$0.29 | 99.18$\pm$1.12 |


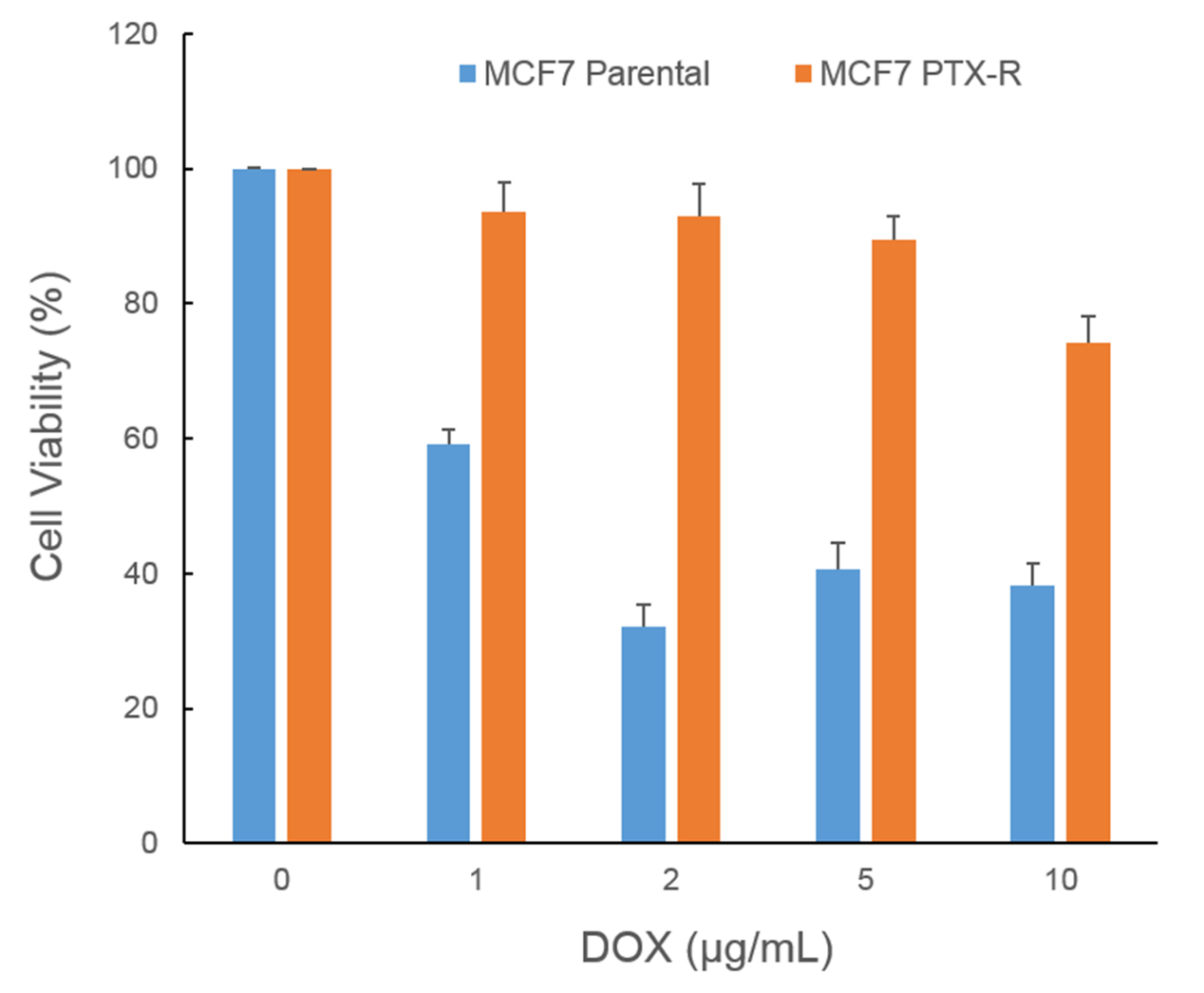
Figure S1. The cell viability of MCF7 Parental and MCF7 PTX-R cells were incubated with free DOX for 48 h. (n=4).


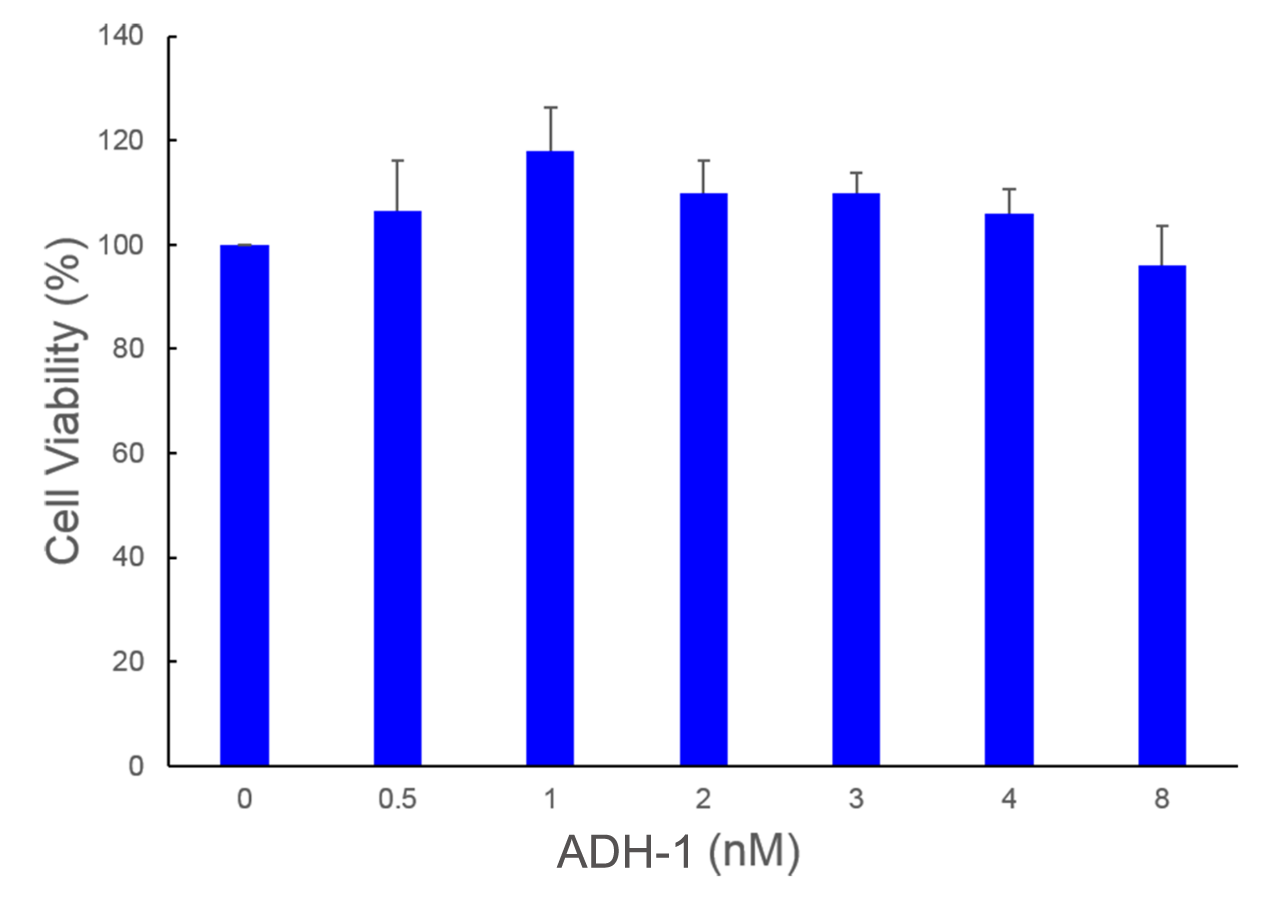
Figure S2. The cytotoxicity of ADH-1 to MCF7 PTX-R cells. (n=4).


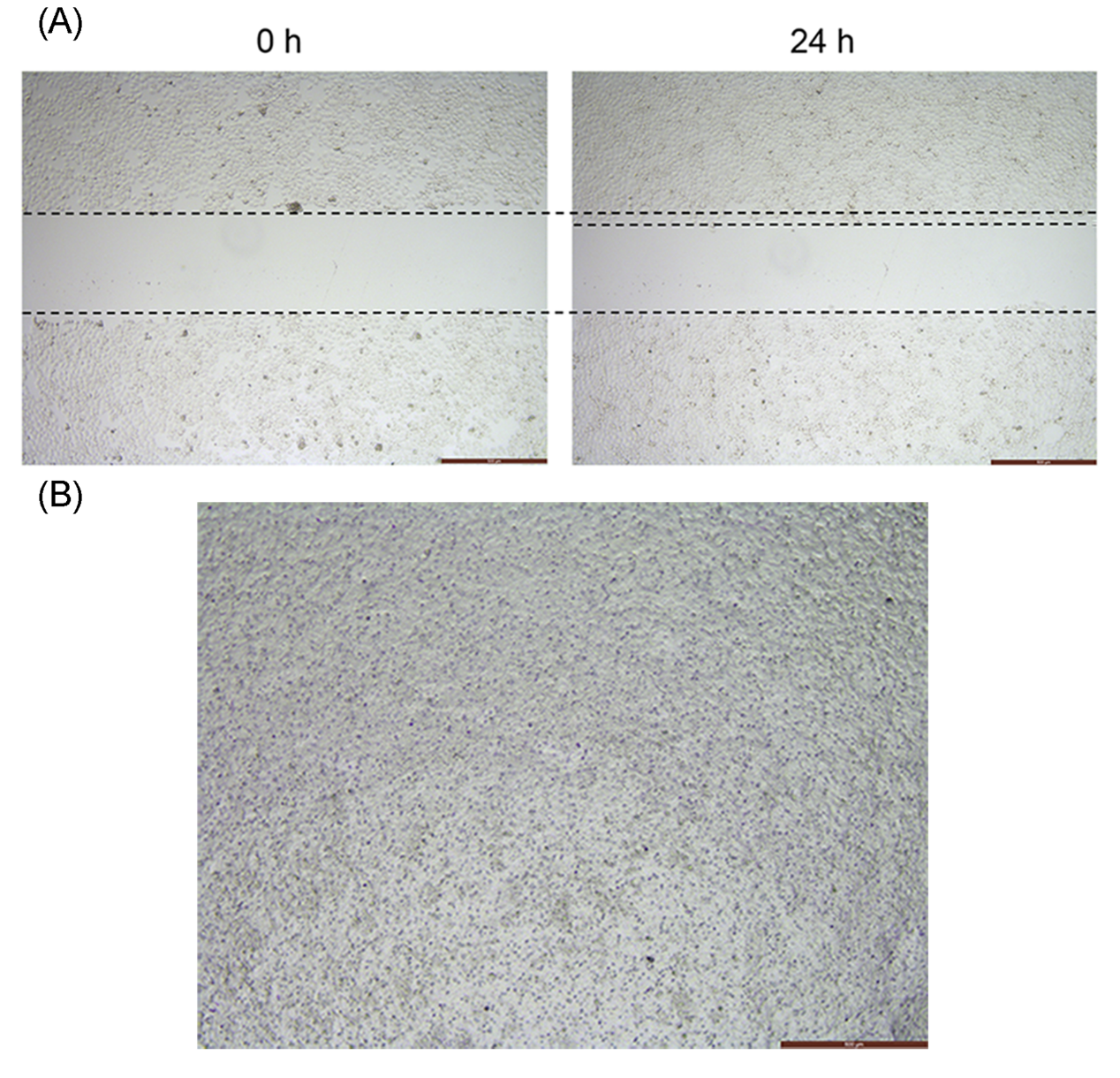


Figure S3. The migration ability of parental MCF7 cells was evaluated by Wound scratch assay (A) and Transwell migration assay (B).
